# Facilitating people living with severe and persistent mental illness to transition from prison to community: a qualitative exploration of staff experiences

**DOI:** 10.1186/s13033-018-0225-z

**Published:** 2018-08-10

**Authors:** Nicola Hancock, Jennifer Smith-Merry, Kirsty Mckenzie

**Affiliations:** 1 Faculty of Health Sciences, University of Sydney, J Block, Cumberland Campus C43J, PO Box 170, Lidcombe, NSW 1825 Australia; 2 Menzies Centre for Health Policy, University of Sydney, Sydney, Australia

**Keywords:** Mental illness, Criminal justice, Prisons, Community integration, Transition, Forensic care

## Abstract

**Background:**

Transition from prison to community is a challenging time for all people who have been incarcerated. It is particularly challenging for those also living with serious and persistent mental illness. This study explored staff experiences and perspectives of what helped and hindered them in their work to support that transition.

**Methods:**

Semi-structured interviews were conducted with 12 mental health staff working across three service sectors directly engaged in the process of supporting people with mental illness transitioning from prison to community; the forensic mental health provider Justice Health, Community Mental Health and a non-government delivered community-based service called Partners in Recovery. Data were analysed using constant comparative analysis.

**Results:**

Five main themes were identified through the analysis. All five themes were key practices that, when occurring, supported staff to work in a way that they felt would maximise positive outcomes for people transitioning from prison to community. These included: housing secured before release; clearly defined and effective communication pathways; shared understanding of systems and roles; in-reach and continuity of contact, and consumers’ pre-release preparation and knowledge. All staff participants described barriers to good transition to community outcomes when some or all of these practices could not, or did not, occur.

**Conclusions:**

Staff experiences highlight the complexity but importance of getting multi-sectorial partnerships and practices right for good prison to community transitions for people living with serious and persistent mental illness. Currently fragmented and disparate systems and practices need to align and clear expectations and understandings need to be shared across the whole. These changes, along with prioritised housing are likely to lead to better long-term outcomes for people.

## Background

People living with severe and persistent mental illness (SPMI) are disproportionately represented within general prison populations internationally [1, 2]. The percentage of NSW prisoners^1^ in 2006 with at least one mental health disorder, including drug and alcohol abuse, was 80% compared with 31% within the general population [3]. Similarly, the 2010 National Prisoner Health Census found that self-reported rates of mental illness in prisons were 2.5× higher than those in the general population [4]. These findings reflect similar rates in international data [5].

Repeatedly, reported outcomes for released prisoners living with SPMI are significantly worse than for their peers without mental illness. Recently released prisoners living with a SPMI are more likely than their peers to be homeless, unemployed, living with other former prisoners or current drug users, experiencing drug and or alcohol misuse issues, and more likely to receive less support from family members when compared to recently released prisoners without mental illness [1, 6–9]. They were also at a much higher risk of suicide [6]. Longitudinal Australian data evidences significantly increased rates of poor longer term outcomes for released prisoners with a self-reported mental illness diagnosis across a multitude of social and health domains, even after adjustment for pre-existing disadvantage [10].

The immediate period after release from prison to community appears to be a critical point in moderating these poor outcomes. It is well evidenced to be a period fraught with challenges and increased risk for people living with severe and persistent mental illness [1]. In transition to community living, former prisoners confront numerous new challenges. These typically include finding somewhere to live, gaining employment, re-connecting with family and previous social networks, or alternatively, trying to refrain from re-connecting with these previous social networks and supports in order to prevent returning to old patterns of behaviour that previously resulted in their incarceration [11]. This then means an additional challenge of establishing new social networks and new daily routines. The burden of SPMI further adds to the complexity and difficulty of successfully negotiating these community re-entry challenges and for many, seeking mental health treatment is not seen as the first priority [12]. Low rates of social support are compounded by poor clinical treatment following release from prison [7] leading to increased risk of suicide [6, 8, 13] and for some entry into a vicious circle of relapse and reoffending [1, 14, 15]. Small studies have shown that recidivism and suicide is reduced in those offenders with mental illness that access pre-release planning and transition support compared to those that do not [16, 17].

In an attempt to improve the prison to community transition experience and thus longer term outcomes for people living with SPMI being released from prison into a large Sydney-based LHD in NSW, a recent collaboration was forged between key stakeholders involved in the transition phase. These stakeholders were: Justice Health, the LHD’s Community Mental Health team and a Partners in Recovery (PIR) program. This collaborative inter-agency working group is called custody to community (C2C). It is important to note that within the Australian state of New South Wales, mental health staff working within prisons are allied-health workers employed through Justice Health rather than directly through the NSW Department of Health. Community Mental Health teams are NSW Department of Health funded, multi-disciplinary allied health teams providing community-based case management and treatment for people living with mental illness. PIR programs are funded by, and thus answerable to, the Australian federal government rather than the state government and the PIR involved in this study is managed by a non-government organisation or charity. Thus, there is complexity in terms of differing lines of accountability across the three stakeholders.

Alongside the C2C initiative, the University of Sydney were engaged to conduct an independent qualitative study to gain an understanding of the current working experiences and perspectives of staff from each of the three organisations and from consumers involved in transitioning from prison to community. This paper reports on staff perspectives on what currently helps or hinders them to successfully support people living with SPMI to transition from prison to community.

## Methods

A qualitative study design was employed in order to gain an in-depth understanding of staff perceptions and experiences given the limited current understanding of the research topic [18]. Methods, informed by a grounded theory approach, included coding, constant comparative analysis, concurrent data collection and analysis and memo-writing. Separate ethics approvals were obtained from the Justice Health and Forensic Mental Health Network Human Research Ethics Committee and the Sydney Local Health District (Royal Prince Alfred Hospital Zone) Review Committee before recruitment commenced.

Written consent for de-identified publication of results was obtained from all participants prior to interviews.

### Sampling and recruitment

Participants met the following criteria: (i) worked within one of the three partnering organisations and (ii) had experience of working with people with SPMI transitioning from prison to community. Staff were informed about the study by members of the C2C team and invited to contact the research team directly if interested in participating.

### Data collection

While reported here in a linear fashion to aid the reader, data collection and analysis were conducted in an iterative, concurrent manner. Semi-structured interviews were conducted after informed consent was obtained from each participant. The authors developed an interview guide with broad questions focusing on key aspects of transition including information transfer, referrals, client and organisational-related barriers and enablers, roles and responsibilities. The guide was designed to be used flexibly so that participants had the opportunity to delve into and further discuss topics or experiences they deemed to be most pertinent. Probing questions encouraged deeper explanations and elaborations. Interviews were either conducted face to face or over the telephone due to participant preference. Interviews took between 14 and 50 min, averaging 34 min. Interviews were recorded, transcribed verbatim, and coded as soon as possible after each interview.

### Data analysis

Data were analysed using constant comparative analysis to compare data within transcripts and between transcripts [18]. Two authors independently coded the first two transcripts, and then met to discuss and find consensus in early coding decisions. Subsequent transcripts were then coded by the third author. Throughout data analysis, regular reflexive discussions between authors ensured emerging codes were representative of the data, enhancing coding rigour [18]. NVivo computer software [19] was used as a data management tool and to facilitate team collaboration throughout the analysis process.

## Results

### Participants

Twelve staff participated in the study including four from each of the Community Mental Health (CMH), Partners in Recovery (PIR) and Justice Health (JH) services. These included seven males and five females. In order to maintain confidentiality within this small community, demographic details of participants are not described in further detail. To further maintain confidentiality, participants are not named, but rather, identified by PIR, JH or CMH (depending on place of work) followed by a number.

### Overview

Five main themes were identified through the analysis. All five themes were key practices that supported staff to work in a way that they felt would maximise positive outcomes for people transitioning from prison to community. These included: housing secured before release; clearly defined and effective communication pathways; shared understanding of systems and roles; in-reach and continuity of contact, and consumers’ pre-release preparation and knowledge. All staff participants described barriers to good transition to community outcomes when some or all of these practices could not, or did not, occur.

### Housing secured before release

An overarching theme repeated throughout and across all interviews was the central importance of housing being organised prior to a person’s release from prison. Enablers and barriers to this optimum outcome occurred both during the incarceration or pre-release preparation period and at the point of release. All participants, regardless of which sector they worked in, repeatedly described housing as the pivotal element of successful release and community transition for the consumers with whom they worked. As CMH4 said, “We know from our client group that as stable arrangements as possible is likely to keep them more well in the community. Housing’s a huge part of that.” Participants described three reasons why they believed that secure housing was central to positive community transition: (1) somewhere to live is the person’s first priority; (2) without housing they are lost to care, and (3) housing helps break a cycle of returning to poor previous relationships and routines.

#### Somewhere to live is the consumer’s first priority

Staff across all three sectors talked about mental health treatment not being consumers’ first priority after release when they were facing more urgent needs. For example, JH10 explained: “when leaving custody, somebody’s main needs are where they’re going to live.” Stability of life circumstances needed to be addressed before mental health support and treatment could become a priority for consumers: “It’s definitely not going to be a priority for somebody to meet up [with a mental health worker or clinician] when they’ve got no place to live, they’ve no food, they’ve no clothes… no access to a phone, no access to money, no ID.” (PIR2).

#### Without housing they are lost to care

Community-based staff from both CMH and PIR described the challenges and failures of trying to locate and connect with consumers who were released to homelessness. Variations of the following story were repeated within the interviews:

“…they’ve released people back into the community without any sort of stable accommodation or follow-up arrangements. We’ve had numerous situations like that. We’re… scrambling around, in the eleventh hour, trying to find somewhere for someone to live so that they’re not lost to care.” (CMH4)

One of the consequences was that they were difficult to locate and connect with for workers to provide support:

“…when they get [released], if they don’t have a mobile phone… how do I contact them? … we don’t know where they’re going to be going because there was no housing arranged… they are released homeless. So… I just have to call around. I call different refuges and see if they are there.” (PIR3)

When people were released outside of the catchment area of the Community Mental Health services that supported them prior to incarceration, housing and continuity of care suffered: “they’re likely to… fall through the cracks if they go outside of the Sydney local health district and they’re our client.” (CMH4).

Housing was a key foundation upon which other needs could be met, offering a stable base upon which to accept mental health-related support. Describing the benefit of housing for maintaining people’s engagement in mental health treatment, one participant said:

“If we’re able to meet them at the place where they’re being released and drive them to an accommodation that we’ve organised … someone would stay there. That gives us a chance to find them more stable arrangements, because that keeps them engaged with the mental health services. We can make sure that they’re having their treatments. We can assess their mental state.” (CMH4)

Finding emergency, temporary housing then becomes the primary initial focus of the worker. Even securing temporary housing enabled mental health workers to start to connect and work with consumers: “Often they [are released] back into homelessness, so we try to support them to find somewhere where we can at least continue to see them until we find them more stable accommodation.” (CMH4) Referring to short term emergency housing, one participant commented “it’s not the most ideal place but at least… he is stable somewhere and we can go and see him in the community and start the process wherever he is at.” (PIR3).

While community-based staff described their frustration with people being released without prior warning and without accommodation organised, Justice Health staff described frustration with this expectation being placed on them when release and housing were often beyond the scope of their roles. As one participant commented: “Community health teams… will ring me up and say, ‘Well we’re not taking them because we don’t have an address.’ Sometimes we say, ‘Well, we don’t know until the patient [prisoner] gets out.’” (JH11)

#### Housing helps break a cycle of returning to previously challenging relationships and routines

A third reason staff identified housing as critical to good post-release outcomes was the opportunity it provided for people to break free of previous unhelpful relationships and routines. Talking about the challenges of needing to rely on old friends or family for accommodation, PIR2 said: “people that are recently released… might start interacting with old friends or old family and re-engage in either criminal activity or be impacted by drugs or alcohol addiction”. PIR 3 said “…we understand that relationships are very important, and that in a lot of the cases the only relationships these people have are not the most helpful towards recovery”.

While housing was the most critical element described by all participants in relation to post-release outcomes, all seemed resigned to the reality that temporary housing was the inevitable and only option for most people released from prison. They talked about consumers having “burnt bridges” (PIR2) with many housing options and about the years long waiting lists for permanent department of housing accommodation: “the reality is that the waiting lists [for housing] are huge.” (PIR1) “We can help with referrals to Housing NSW but no one gets housing accommodation straight from jail” and “one of our biggest issues is the lack of accommodation” (JH7). The system for getting accommodation was not proactive with limited action taking place prior to the time of release. One justice health said “you have to be released and then [the person being released] go[es] to the office and ask[s] for emergency accommodation” (JH7).

### Clearly defined and effective communication pathways

Participants all talked about the necessity of having strong and clearly defined communication pathways for facilitating an individual’s transition to the community. For example:

“…[we] need to have that ongoing, open line of communication and [to] share… information for [staff] to be able to either implement support or develop an understanding about what’s going on for that person to better support them.” (PIR2)

However, invariably participants described difficulties accessing the information they needed from other sectors in order to do their job. PIR2 said:

“Just… finding out who you’ve got to talk to, even organising something like that. It can be really challenging to track down the appropriate person because you still have to send through requests to do that. It has to be signed off by the manager of security, you need to identify yourself. It’s not just [about] ringing up and going ‘Hey, I want to talk to this person’. So yeah, that can be quite a convoluted confusing process”.

Staff from all sectors talked about those in other sectors not returning their calls or email messages. For example, PIR1 said: “[the] biggest barrier would be getting in contact with these people and for [them] to return our calls and answer our inquiries.” and JH7 said: “First of all, they hardly ever call you back.”

In the absence of clear pathways of communication, staff described relying on prior working relationships with staff in other organisations and calling on favours. Staff relied on their pre-existing relationships to find out about release dates, and where clients were in the system or what services they were receiving. “As it happens… [one staff member] used to work … here as a nurse on the team, and so she’s really accessible. She’ll call us when she knows of somebody is possibly being released” (CMH3). This reliance on previous working relationships to overcome a perceived reluctance to share information was also the experience of forensic staff:

“it’s a lot easier if I know the person… [I was] working in health for so many decades. I do get on the phone to people that may know me and it’s a lot easier… I may have the patient’s name, date of birth, and a local address and they’re still a bit reluctant to give me information [if they don’t know me]… networking is probably one of my best tools.” (JH11)

In the absence of these informal pre-established connections, staff indicated that access to information was dependent on “who you got on the day” (PIR3). “Oh it depends who you’re dealing with. It’s so idiosyncratic. I mean some people are fantastic. Other people are brutally indifferent. It just isn’t consistent” (CMH6). They described a process of repeatedly making contact with the service until connecting with someone prepared to share information: “One of the things that [a senior staff member]… said [was] ‘Look, just ring them tomorrow and you’ll get someone else’” (CMH5). This sense of powerlessly hoping to reach a helpful individual was frustrating for staff:

“It should not be like that. It should be standard. Whoever it is behind supporting these people before release within the justice system, I think everyone in there should be doing the same thing. Coordination and collaboration, it always helps a lot.” (PIR1)

As well as struggling to access information, staff talked about problems working out who to share important information with. CMH5 provided some examples:

“my guy’s gone into jail, and… he’s a diabetic, and I want to let them know about that. If I could figure who to talk to … [it would be] beautiful…. [or] a person going in who is on a depot. If I can get to the right person, tell the guys over in [prison], ‘Here’s what his treatment history is, current medication, his last depot date,… his side effect profile’ and so on, [that] would be very nice.” (CMH5)

In direct contrast, JH11 talked about the barriers to getting this same urgent information from the Community Mental Health team when someone in prison was becoming unwell:

“it makes it very difficult, you’ll have to wait for [a Release of Information authority] to come back and this patient has become unwell but [CMH] can’t tell me exactly what [medication] they’ve been on, what allergies they’ve got, then it makes it very difficult to prescribe a medication.” (JH11)

Participants repeatedly discussed communication problems around dates for court hearings and potential release dates. Repeatedly community-based staff discussed the challenges and frustrations of not being able to find out or not being informed about release dates of people with whom they worked: “people’s release dates change constantly and people aren’t informed and people don’t know. That could be family… [or] support providers… even parole don’t know a lot of the time” (PIR2). Staff within the prison system itself were often equally unaware of when people were or actually had been released and thus unable to pass on information expected of them: “Somebody has a release date, they’re released by Corrective Services and we don’t always get to know about it. So that’s one of the difficulties for us.” (JH10).

To address these barriers around communication pathways, participants, described a desire for more systematic, consistent and effective approaches and policies to the transfer of information between sectors:

“Whatever the minimum standard of information transfer is, needs to be identified and published.” (CMH6)

“we’ve got about ten clients that have been in and out of jail the last few years, and every experience is different. There’s not a consistent approach … [A] consistent process about how to receive the information would be good. Either we devise something with people, or they let us know how they give us that information or how we can find out that information.” (CMH4)

CMH5 suggested “a website [that] you could go to and you could find out who you talk to for particular issues at a particular location”. JH11 expressed the desire for an integrated online information system:

“I know other health organisations [where] everything is done on database, progress notes, is all done on computer. So if I want to find out how a patient was … I can go online and find it. The same sort of system would be great here, I could log in and… know what’s going on with this patient [prisoner]”.

These information systems suggested by interview participants were desirable because they bypassed the need for often problematic or inconsistent connections with individuals and organisations mentioned above.

### Shared understanding of systems and roles

Perhaps also stemming from the unclear or ad hoc communication pathways described above, staff within each of the three sectors repeatedly talked about staff from the other two sectors not valuing their role, not being clear about what their role was, or not understanding the limits to their role. Variably expressed, all participants said things like: “they [staff working in other services] don’t understand the process.” (JH7). They expressed the belief that other agencies did not understand either the central role of their organisation, or the limitations inherent within the systems within which they worked. Staff felt that this lack of clarity put expectations on them which were difficult to meet. The role of PIR, the most recent service or program in the system, was particularly confusing for staff from other sectors. CMH6, indicated that they needed “a clearer sense of what Partners in Recovery could or could not provide”. PIR8, speaking about a Justice Health staff member said, “I think he might have been a bit disappointed that Partners in Recovery were not a direct support service”. Grappling with the PIR role, JH10 said: “So we’ve certainly tried to refer on, is my understanding, but … their [PIR] main role is around improving communication between services who are looking after a client, is my understanding. So maybe not as useful in that immediate, for that immediate transfer of care upon release.” In contrast, JH12 continued to refer to PIR for housing assistance: “We contact Partners in Recovery when usually patients don’t have accommodation—will need some help with accommodation and follow up”. However, PIR participants talked about an unrealistic expectation that others had around their capacity to arrange last minute accommodation”

This lack of understanding was not just about roles of staff within different sectors. It also extended to the practical or systemic barriers that existed within service systems, particularly Justice Health which was constrained by working within a high-security environment.

“We don’t have access to a phone, we don’t have mobile phones… Communication is not always the easiest to do and people that have never worked in the jail don’t understand what it’s like to try to communicate between somebody working within the confines of a secure environment like a jail to outside” (JH11).

“I’ve had Community Mental Health teams on the phone asking, ‘Well, when was the last time they had their medication, their depot?’ … I go, ‘I don’t know… joint records is outside this jail and I have nothing to do with joint records. I’ll give you a telephone number or whatever’.” (JH11)

“They [community-based staff] don’t understand the process. If someone was released from court yesterday, I could not possibly do a referral the day before, because we don’t know that the person will be released and they will often say, ‘Well, why didn’t you do the referral earlier?’” (JH7)

Multiple practical impacts resulting from this lack of role clarity were discussed by participants. One of the most frequently discussed challenges was maintaining contact with people once incarcerated, or establishing contact with them prior to release.

### In-reach and continuity of contact

While particularly emphasised by community-based staff (those from PIR and CMH), all participants talked about in-reach being a critically important element of successful transition to the community. In-reach involves community-based staff entering the prison setting to meet with consumers prior to release. This might be new referrals where release is anticipated or pre-existing clients with whom they had already engaged in the community prior to incarceration. In-reach was seen as critical for establishing rapport, understanding and preparing for individual needs, and maintaining contact with people they already had a support relationship with. Participants described both procedural and attitudinal barriers to in-reach.

#### Establishing rapport and understanding individual needs

Opportunity to develop a positive therapeutic relationship with the person in prison prior to their release was reported to increase the likelihood of maintaining contact and engagement with the person after release. “If we meet someone while he or she is in jail, the rapport is established…it is more likely that they engage with us in there [prison]” (PIR1).

In-reach also enabled staff to understand and thus prepare for the individual needs of the person. Describing the value of in-reach, PIR2 said it enabled them to “talk… a bit about what they want to do, how we can support them.” and CMH5 said:

“when I went and met with him and could sit with him, and could have him tell me how difficult it was in that place, I could [understand] how he got there… what had happened and how we could do things differently next time. All that stuff was an important marker, an important punctuation point in his treatment experience.”

#### Maintaining contact

Community Mental Health staff particularly talked about the value of regular in-reaching and continuity of contact over an extended period of time with people that they had an established, long-term, therapeutic relationship with prior to incarceration.

“We’ve been the only sort of support for them for really long periods of time, and we want to know, that they know, that we’re still here when they leave. So it’s about maintaining therapeutic alliance with the consumer that’s been taken into custody.” (CMH4)

CMH5 explained that before a person with whom he worked was in jail, “We had a good relationship he and I and”, because of regular in-reach “we still do”. As these quotations show, maintenance of long-term therapeutic relationships was viewed as central to ensuring ongoing connection and treatment once the individual transitioned back into the community.

#### Procedural barriers to in-reach

While valued, in-reach was almost universally described as problematic to achieve or carry out. Participants talked about “the hassle” (CMH5) and “the hoops that you’ve got to jump through to see people” (CMH4).

Participants working in community contexts repeatedly talked about the time-consuming and arduous process of getting clearance and approval to enter the correctional facility. “Even finding out who you’ve got to talk to [to get access], it can be really challenging to track down the appropriate person… that can be quite a convoluted confusing process” (PIR2). PIR1 said that the process of getting approval or clearances completed to “get access… can take 4 weeks”. However, even after the paperwork had been completed, approval to enter the facility gained, and arrangements to visit made, further barriers exist.

Community workers from both PIR and Community Mental Health both described arriving at the correctional facility and facing repeated challenges to “get[ting] through the doors.” (CMH4). Participants relayed stories of themselves or colleagues arriving at the facility as pre-arranged only to be turned away. This sometimes happens on multiple occasions before they can see someone: “We’ve driven to the facility, we’ve shown up two or three times before we actually get to see the person. [It has taken] over 3 or 4 weeks” (CMH4).

The main front desk or reception related barrier involved staff in the entrance area not having any record of the planned appointment or not knowing how to contact staff who were meant to be facilitating the visit.

“We’d talked to [justice health staff] about going to see people in custody and [then when] we show up and they didn’t know that we were coming, and we’ve been turned away at the door. That’s happened several times as well.” (CMH4)

“I submitted the approvals, faxed through my deeds and everything 2 weeks before. Organised the date to come over, I get there, it’s four in the afternoon, [and the front desk staff said], mate, sorry, I don’t know what you’re talking about. I haven’t seen that form. I can’t find it.” (CMH5)

In the second instance related here, the worker left without entering the facility. One participant talked about the front desk not having any record of the corrections staff member who worked with the person they were coming to visit: “Thank God I came prepared. I had pre-notes… trailed emails with the Corrections key coordinators there… I went to the booth where you get scanned… and they had no idea who this person was who works with them… they didn’t even know that staff existed” (PIR3). Again, the PIR worker left without being able to access the person they had come to visit.

Other barriers faced at the front desk were related to internal security events: “…you set up a time for the NGO provider to come in and they get in and find the Correctional Services have locked the place down because of an incident.” (JH10)

#### Attitudinal barriers to in-reach: why are you still coming?

Community Mental Health staff talked about being actively discouraged from on-going contact with people they had been working with prior to incarceration. CMH4 said “We’ve had lots of contacts and conversations with people where we just [said] ‘look, we just want to see them and say hello to them. It took us a really long time to get to this point, we just want to keep that relationship’. But we’ve actually been given some timeframes where we can come and see them a couple of times, but after that correctional staff say ‘there’s no real need for you to keep coming’”. Talking about a specific client CMH4 continued to say “We went and saw her a couple of times after that, they [correctional staff] said ‘well, that’s pretty much enough don’t you think?’”. In direct contrast to this, a staff member from Justice Health said “I can only say positive things about them [PIR staff] because they’re always willing to come and meet clients here which never happens in a Community Mental Health team… they’re not interested.”(JH7)

### Consumers’ pre-release preparation and knowledge

A lack of appropriate or adequate preparation of people prior to their release was also repeatedly discussed in the interviews. Preparation included: knowledge and understanding of the support system post-release; basic living skills and drug and alcohol rehabilitation; and an individualised approach to release and resources.

#### Knowledge and understanding of the system

Poor understanding and communication between agencies also led to mis-communication to people being released about the various roles of services and their capacity to support them post-release. This is encapsulated in the following quotation:

“This is one of the reasons why… the preparation from before they are released needs to be strong. I have had consumers coming to us asking ‘What are you going to do for me?’,… They become disappointed because they have been told [by correctional staff] that once they get out [PIR are] going to get you a house, they’re going to get you on the pension, they’re going to get you this, they’re going to get you that. That is not realistic.” (PIR1)

Staff from across all stakeholder groups felt that people being released were not well prepared for the limited capacity of services, particularly in relation to securing housing.

“I think the hardest bit is that no one, before these people are being released, works with them on a one-to-one basis, encouraging them to understand that once they’re released they’re on their own very much. They will have the support but the supports don’t guarantee that they’re going to go into public or community housing.” (PIR1)

Referring to people’s expectations of them whilst in prison, JH7 said:

“… but no one gets housing accommodation straight from jail. You have to be outside the jail. You have to be released and then they have to the go to the office and ask for emergency accommodation and then follow the process, whatever the process is.” (JH7)

#### Basic living skills and drug and alcohol rehabilitation

Community-based staff talked about the need to better prepare people who had been in prison for extended periods for what had changed outside of the prison walls. One participant spoke about this at length:

“Yeah, the biggest factor and the biggest learning experience for me as well [is witnessing peoples] massive anxiety [when released]… They’ve told me ‘it’s like it’s a brand new world for me!’ They… went in 10 years ago. ‘What kind of a phone is that? It has no buttons!’ …another [person], he got out of jail [after] 17 years. He was released, with no clothes, nothing. So I [said] ‘Okay… let’s go get some clothes. Are you okay with Kmart?’ He had nothing, so I get a basket and even walking into Kmart… he’s a big guy, he’s a big giant guy [but] he [said] ‘I’m feeling anxious.’ I [said] ‘Okay just focus on my voice. Let’s take a seat. Let’s do some breathing exercises. How do you feel? Cool. Let’s go back. How are you feeling?’ Just doing all that stuff and then going to the counter, he [asks], ‘What do I do?’” (PIR3)

Several participants discussed this need to address the loss of living skills and confidence that occurs during extended periods of incarceration. Advocating for programs prior to release to facilitate adjustment to life back in the community, PIR8 said: “Just dealing with that whole anxiety of stigma and the practicalities of life apart from the mental health… I think training [prior to release is needed]. I’d love that.”

Beyond these challenges stemming from a loss of living skills and new ways of doing in the community, a couple of participants suggested that people still had access to illicit substances whilst in prison. This created an additional barrier to their successful transition to community:

“You’re not supposed to have access to substances when you’re in there. We know that that is not always the case. In a lot of the cases what we can see is that the person has not undertaken any rehabilitation when it comes to substances” (PIR1).

## Discussion and conclusions

The experiences of all 12 staff across the three service sectors portray a fragmented rather than well integrated system. Participant’s all spoke passionately about the desire to achieve better outcomes for people living with mental illness transitioning out of prison. They also all described an array of barriers, stemming from lack of overarching system leadership, which impeded their ability to do so. Participants repeatedly spoke about: others not understanding what they do; not knowing who to speak to or connect with in other service sectors; a lack of clear or transparent systems or procedures. On top of these systemic work-place barriers, housing options for people in the precarious period of prison to community transition were almost invariably not available. People were typically released to homelessness. When housing was secured, it was transient emergency accommodation rather than stable and secured. This fragmentation and lack of housing resulted in Community Mental Health and support services loosing contact with consumers at this critically important time of transition. The fragmentation and lack of housing thwarted staff attempts to enhance people’s transition from prison to community. These findings are reflective of the broader international literature.

To enhance the capacity of staff across service sectors to successfully support people living with SPMI to transition from prison to community in New South Wales leadership is needed. Specifically, leadership and a commitment to the following actions, supported by best evidence available, is necessary:

First, shared information systems, ideally electronic, along with clear, agreed upon, handover practices including key contact people would greatly enhance service integration. Handover practices need to include basic clinical and support information along with routine and early notification of court hearings dates for people on remand and dates for parole board hearings for people who have been convicted. Both should be available and shared well ahead of time or available on a shared information system with proactive access systems established. Poor information sharing has been identified as a major obstacle in otherwise well-established transition support programs [20]. Various systems have been devised to establish appropriate approaches to support transition from prison to community [21–23], however these systems are ineffective without an overarching connection between all jurisdictions involved.

Second, establishing sector-bridging communities of practice at both state and local levels to enhance shared understanding of roles, responsibilities and challenges would also enhance system integration. Networks or communities of practice surrounding all elements of forensic (prisons and forensic hospitals) mental health related care have been developed in a number of different countries to deal with these issues [23, 24]. These include the Justice Health Collaboration project in the USA [21] and the Forensic Mental Health Services Managed Care Network (Forensic Network) in Scotland [24]. The Forensic Network was developed in order to establish an educated and connected workforce to manage forensic mental health care and treatment, including transition support. It provides networking, training and has established care pathways and other administrative reforms [25]. It works at both a state level and develops local projects to reform practice where needed. A system such as this would address many of the problems of poor understanding and communication in the NSW context.

Third, as a part of establishing agreed upon practices, in-reach of community-based staff to visit and connect with people prior to release needs to be standard and expected practice. Again, this is supported by the international literature and is shown to be effective [14, 26–28].

Fourth, a stronger focus upon preparing people for release to community living is needed. This program would build the knowledge and living skills needed to prepare people for how life has changed in the community since they were incarcerated as well as minimising loss of daily living skills through long periods of institutional living. A lack of focus on mental health rehabilitation and community preparedness within prison systems is reported within the literature [29–31]. While a number of prison programs are targeting these needs [32, 33] few evaluations of effectiveness appear in the literature. Alongside these living skills, participants in the current study recommended in-prison drug rehabilitation programs so that individuals were able to start their community-living transition without the compounding factor of a drug addiction.

Finally, and perhaps most importantly, the development of a comprehensive accommodation strategy for people living SPMI being released from prison is essential. This needs to focus on housing stability and to clearly identify who within the complexity of this system is responsible. A plethora of international evidence demonstrates that secure housing is a strong confounding factor in successful transition and that housing established prior to release is thus key to positive outcomes for this vulnerable population [7, 30, 34].

This study was conducted within the Australian State of NSW and while findings appear synonymous with those described internationally, they need to be read with context in mind. There are other service providers working with many people living with SPMI who are transitioning from prison to community. These include, for example, homelessness programs, accommodation provider services and drug and alcohol programs. Staff from these programs might have different or additional insights to share. Further, and importantly, the findings in this study are based upon experiences and perspectives of staff only. The actual people using these services to support their transition may well highlight different challenges or experiences.

## Abbreviations

JH: Justice Health
CMH: Community Mental Health
PIR: Partners in Recovery
C2C: custody to community

## Footnotes

1 We use the term ‘prisoners’ or ‘people in prison’ in this paper while acknowledging that this term is being used to cover people who may be in prison, on remand or in some other criminal-justice related institution.
